# Novel ZnO:Ag nanocomposites induce significant oxidative stress in human fibroblast malignant melanoma (Ht144) cells

**DOI:** 10.3762/bjnano.6.59

**Published:** 2015-02-26

**Authors:** Syeda Arooj, Samina Nazir, Akhtar Nadhman, Nafees Ahmad, Bakhtiar Muhammad, Ishaq Ahmad, Kehkashan Mazhar, Rashda Abbasi

**Affiliations:** 1 Nanosciences and Catalysis Division, National Centre for Physics, Quaid-i-Azam University campus, Islamabad, Pakistan; 2 Department of Chemistry, University of Hazara, Mansehra, KPK, Pakistan; 3 Department of Biotechnology, Faculty of Biological Science, Quaid-i-Azam University, Islamabad, Pakistan; 4 Institute of Biomedical and Genetic Engineering, G-9/1, Islamabad, Pakistan; 5 Accelerator Lab, National Centre for Physics, Quaid-i-Azam University campus, Islamabad, Pakistan

**Keywords:** cancer therapy, cytotoxicity, photo-oxidation, ZnO:Ag nanocomposites

## Abstract

The use of photoactive nanoparticles (NPs) such as zinc oxide (ZnO) and its nanocomposites has become a promising anticancer strategy. However, ZnO has a low photocatalytic decomposition rate and the incorporation of metal ions such as silver (Ag) improves their activity. Here different formulations of ZnO:Ag (1, 3, 5, 10, 20 and 30% Ag) were synthesized by a simple co-precipitation method and characterized by powder X-ray diffraction, scanning electron microscopy, Rutherford back scattering and diffuse reflectance spectroscopy for their structure, morphology, composition and optical band gap. The NPs were investigated with regard to their different photocatalytic cytotoxic effects in human malignant melanoma (HT144) and normal (HCEC) cells. The ZnO:Ag nanocomposites killed cancer cells more efficiently than normal cells under daylight exposure. Nanocomposites having higher Ag content (10, 20 and 30%) were more toxic compared to low Ag content (1, 3 and 5%). For HT144, under daylight exposure, the IC_50_ values were ZnO:Ag (10%): 23.37 μg/mL, ZnO:Ag (20%): 19.95 μg/mL, and ZnO:Ag (30%): 15.78 μg/mL. ZnO:Ag (30%) was toxic to HT144 (IC_50_: 23.34 μg/mL) in dark as well. The three nanocomposites were further analyzed with regard to their ability to generate reactive oxygen species (ROS) and induce lipid peroxidation. The particles led to an increase in levels of ROS at cytotoxic concentrations, but only HT144 showed strongly induced MDA level. Finally, NPs were investigated for the ROS species they generated in vitro. A highly significant increase of ^1^O_2_ in the samples exposed to daylight was observed. Hydroxyl radical species, HO^•^, were also generated to a lesser extent. Thus, the incorporation of Ag into ZnO NPs significantly improves their photo-oxidation capabilities. ZnO:Ag nanocomposites could provide a new therapeutic option to selectively target cancer cells.

## Introduction

Zinc oxide (ZnO) nanoparticles (NPs) exhibit an excellent photo-oxidation activity [1] and are considered as potential photoactivated NPs suitable for clinical applications [2–3]. Photoactive NPs provide an attractive, non-invasive alternate treatment option for cancer [4–5]. It involves the administration of photosensitizers followed by the illumination of cancer-affected areas with light of an appropriate wavelength, and thus exciting the photosensitizer to produce reactive oxygen species (ROS) such as singlet oxygen (^1^O_2_) and hydroxyl radicals (HO^•^) [6–7]. Photo-oxidation holds promises for the targeted treatment and controlled elimination of cancer cells [8]. ZnO NPs have also shown photo-oxidative anticancer activity against different cancer cell lines in vitro [1,9–10].

ZnO NPs have a vast range of biological applications because they are biocompatible, considered to be safe [11] with a survival lifetime of a few hours in the body, and they can be dissociated and absorbed quickly. ZnO NPs exhibit antibacterial properties [12–13], are used in the cosmetics industry [14–15], and are used as nanoscale biosensors [11] and as drug carriers [16–17]. These NPs are being increasingly recognized due to their differential activity against tumor cells while being non-toxic to normal cells [18–22]. The preferential target of ZnO NPs are rapidly growing cells whereas quiescent cells are not as sensitive [14,17,23] and in combination with photo-oxidation a targeted elimination of the cancer cells can be achieved.

The photonic efficiency of ZnO NPs is however considered unsatisfactory, as their photocatalytic decomposition process is slow and needs to be improved [24]. Therefore, it is interesting to enhance their photocatalytic ability and anticancer activity by forming nanocomposites with other materials, including metal ions such as silver (Ag) or iron (Fe) ions [25]. The ZnO:Ag nanocomposites exhibit an improved photocatalytic activity [26–27] and photostability [28] compared to the ZnO NPs. Nanoscale Ag^2+^ itself exhibits antimicrobial and anticancer activity [29], therefore it might be a very interesting and useful addition to the ZnO NPs as it not only enhances the photocatalytic activity of the particles but might also improve their anticancer effects.

In this study, we investigated ZnO and different ZnO:Ag (1–30%) nanocomposites with regard to their cytotoxic effect in human malignant melanoma cells (HT144) and normal cells (HCEC). Combined with the MTT and sulforhodamine B (SRB) assay the cytotoxicity in vitro and the differential effect of different Ag contents in the ZnO nanoparticles affecting cell proliferation was analyzed. The photo-oxidation-mediated cytotoxicity of different NPs was investigated by irradiating the samples with daylight or keeping them in the dark. The nanocomposites were studied regarding their ability to generate ROS and lipid peroxidation by chemical trapping method (DPBF; 1,3-diphenylisobenzofuran) and the thiobarbituric acid-reactive species (TBARs) assay, respectively. Generation of various ROS species was studied by using scavengers such as mannitol, sodium azide (NaN_3_) and dimethyl sulfoxide (DMSO). To the best of our knowledge, the preparation and characterization of ZnO:Ag nanocomposites with varying amounts of Ag incorporated and the study of their anticancer activity has not been reported, yet.

## Results

### Characterization of nanocomposites

**Figure 1:**
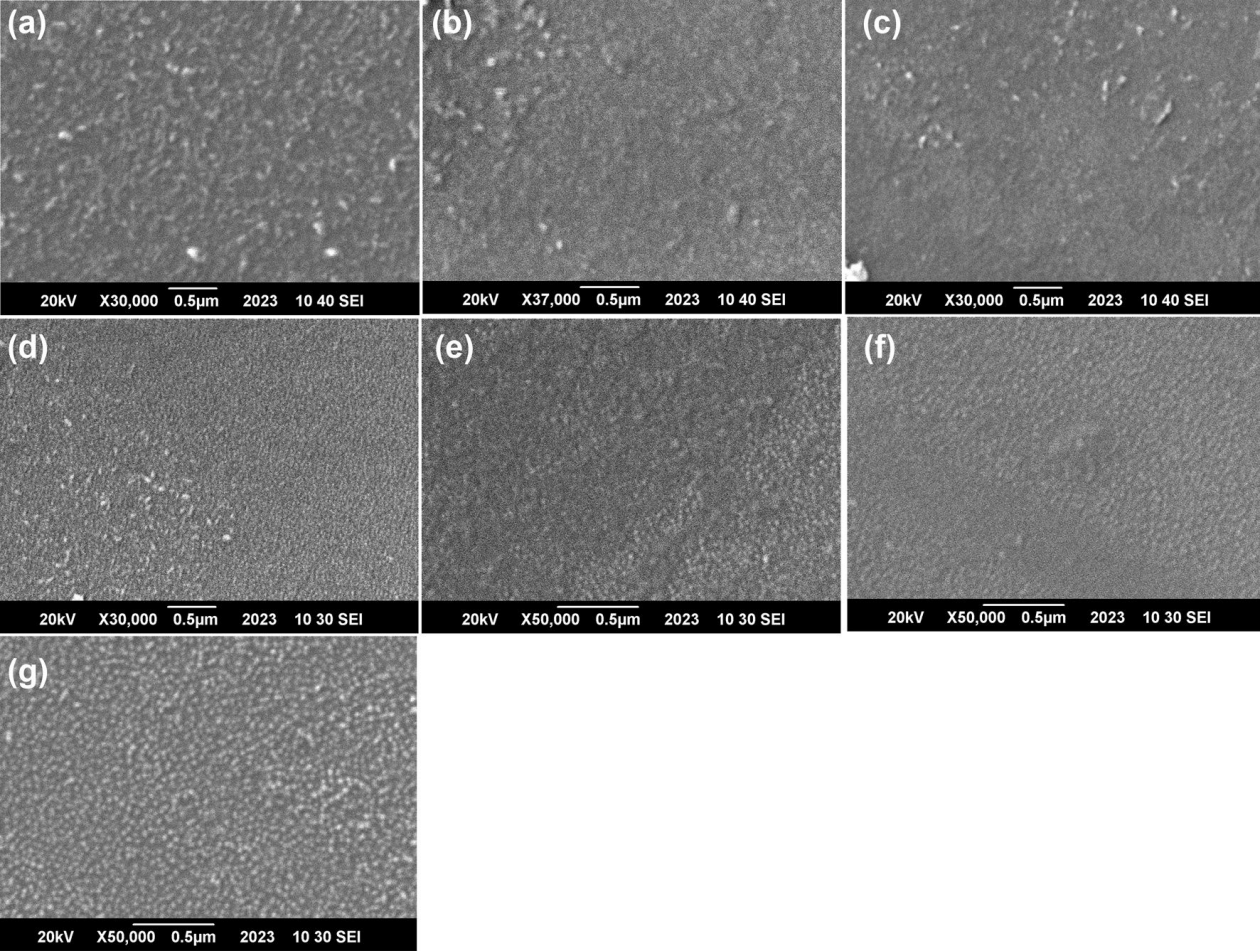
SEM images of the different zinc oxide nanoparticles. (a) ZnO, (b) ZnO:Ag (1%), (c) ZnO:Ag (3%), (d) ZnO:Ag (5%), (e) ZnO:Ag (10%), (f) ZnO:Ag (20%), and (g) ZnO:Ag (30%).

The scanning electron microscopy (SEM) image (Figure 1) shows that all the synthesized composites had nanoscale dimensions, with sizes ranging from 30 to 40 nm.

**Figure 2:**
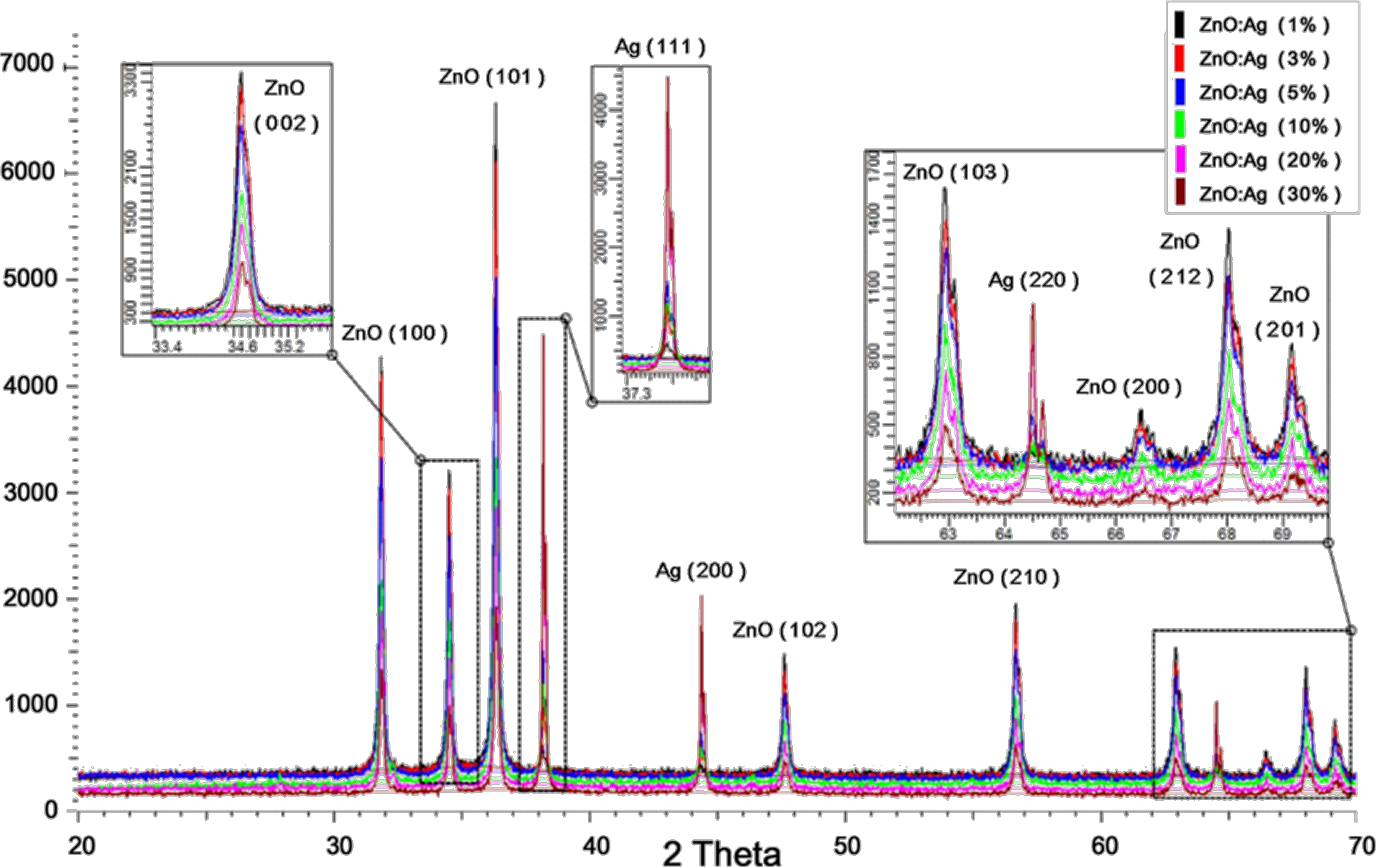
XRD patterns of different types of ZnO:Ag nanoparticles show the ZnO wurzite hexagonal crystalline structure and presence of the cubic crystalline form of Ag. Inset: magnified part of the spectrum showing the decrease in height of the ZnO peaks with an increase in the appearance of the Ag phase.

The X-ray diffraction analysis (XRD) patterns of the ZnO:Ag nanocomposites (Figure 2) contains ZnO phases corresponding to the wurzite structure at 2θ values of 31.8, 34.4, 36.3, 47.6, 56.6, 62.9, 66.4, 67.9 and 69.1° in accordance with the zincite stick pattern COD 9004180. No other peak for the cubic phases of ZnO or any other ZnO structures such as ZnO_2_ or Zn(OH)_4_ was seen. The Ag present in the ZnO:Ag composite nanoparticles appeared as cubic phases of pure silver crystals at 2θ values of 38.2, 44.4, 64.5° in accordance with reference stick pattern COD 9011607. The appearance of Ag as a separate crystal indicates that Ag is not incorporated into the wurzite structure of ZnO but preserved its crystalline form. The co-growth of ZnO wurzite and Ag cubic structures took place through the adopted in situ doping procedure. The decrease in ZnO peak heights with the increase in Ag amount indicated that ZnO crystal structure deteriorated to smaller crystallites as silver started growing as a separate phase along the ZnO crystals.

**Figure 3:**
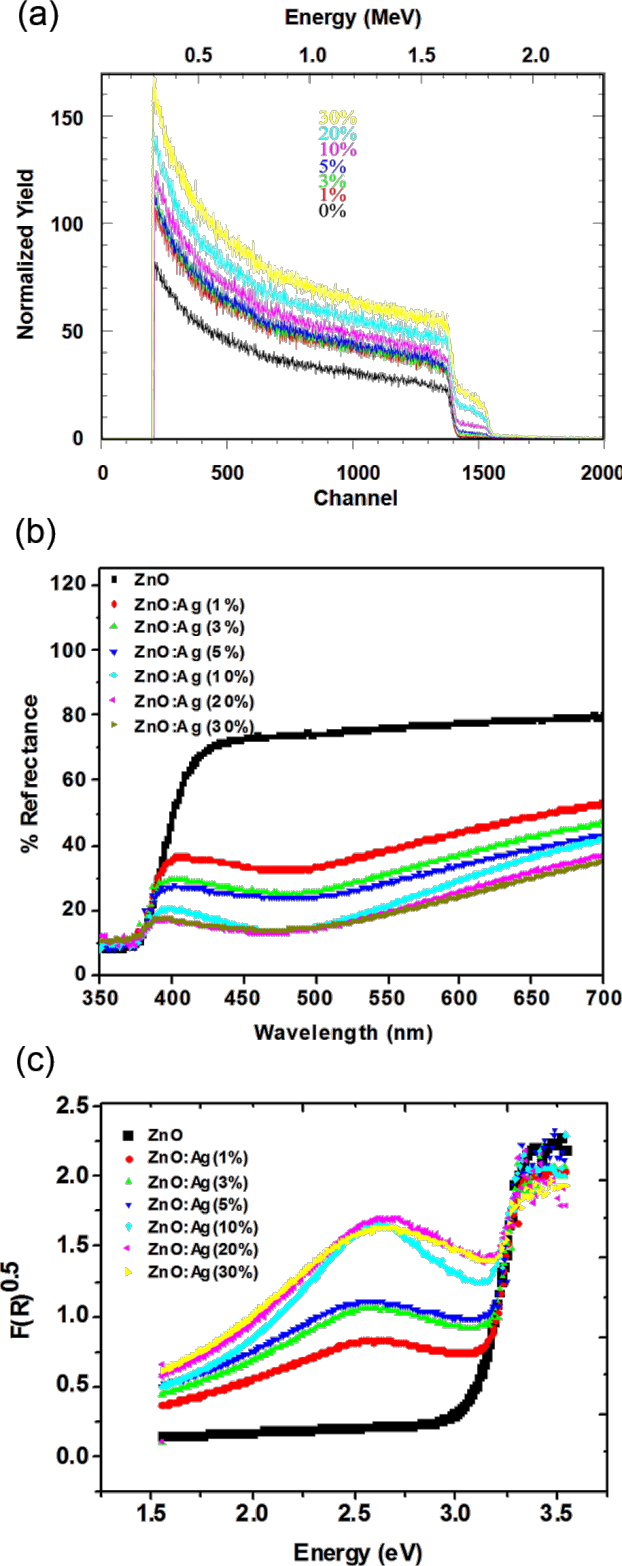
Characterization of the different ZnO and ZnO:Ag nanocomposites. (a) RBS analysis; atomic composition graph is showing the relative amounts of Zn, O and silver, (b) diffused reflectance spectra, (c) band gap energies of the different nanocomposites.

Rutherford backscattering spectrometry (RBS) analysis (Figure 3a and Table 1) shows that our samples contain the correct elemental compositions regarding Zn, O and Ag and confirm the presence of Zn:O:Ag in 1:1:0.01, 1:1:0.03, 1:1:0.05, 1:1:0.1, 1:1:0.2 and 1:1:0.3 ratios in ZnO:Ag (1%), ZnO:Ag (3%), ZnO:Ag (5%), ZnO:Ag (10%), ZnO:Ag (20%) and ZnO:Ag (30%) percent, respectively. Ag was found in fractions of 1.3, 2.9, 4.6, 9.7, 20.6 and 29.3% in ZnO:Ag (1%), ZnO:Ag (3%), ZnO:Ag (5%), ZnO:Ag (10%), ZnO:Ag (20%) and ZnO:Ag (30%) samples, respectively.

**Table 1:** RBS analysis of ZnO:Ag nanoparticles indicating the relative quantities of Zn, O and Ag in the prepared samples.

	Zn	O	Ag

ZnO	1	0.9898 ± 0.0016	0
ZnO:Ag (1%)	1	0.9706 ± 0.0028	0.0132 ± 0.0004
ZnO:Ag (3%)	1	1.0139 ± 0.0110	0.0287 ± 0.0006
ZnO:Ag (5%)	1	1.0163 ± 0.0001	0.0459 ± 0.0027
ZnO:Ag (10%)	1	1.0028 ± 0.0121	0.0973 ± 0.0017
ZnO:Ag (20%)	1	1.0195 ± 0.0212	0.2059 ± 0.0064
ZnO:Ag (30%)	1	0.9938 ± 0.0116	0.2932 ± 0.0002

The band-gap studies of the nanocomposites carried out through diffused reflectance spectra (DRS) analysis show a characteristic absorption edge near 390 nm (Figure 3b). The non-doped ZnO showed a high reflectance, while Ag-doped ZnO showed low reflectance and more absorbance in the visible region. Moreover, the intensity of reflectance decreased with the increase of Ag contents. The band gap energies of the non-doped ZnO and Ag-doped ZnO were calculated by plotting the square of the Kubelka–Munk function [*F*(*R*)]^1/2^ versus the energy in eV (electron volts) [30].

A band-gap decrease was observed in all the Ag-doped ZnO to the varying extents depending upon the doped Ag (1, 3, 5, 10, 20, and 30% Ag). Silver resulted in a band structure in visible region in all the ZnO:Ag nanocomposites (Figure 3c).

### Screening of NPs for cytotoxicity

**Figure 4:**
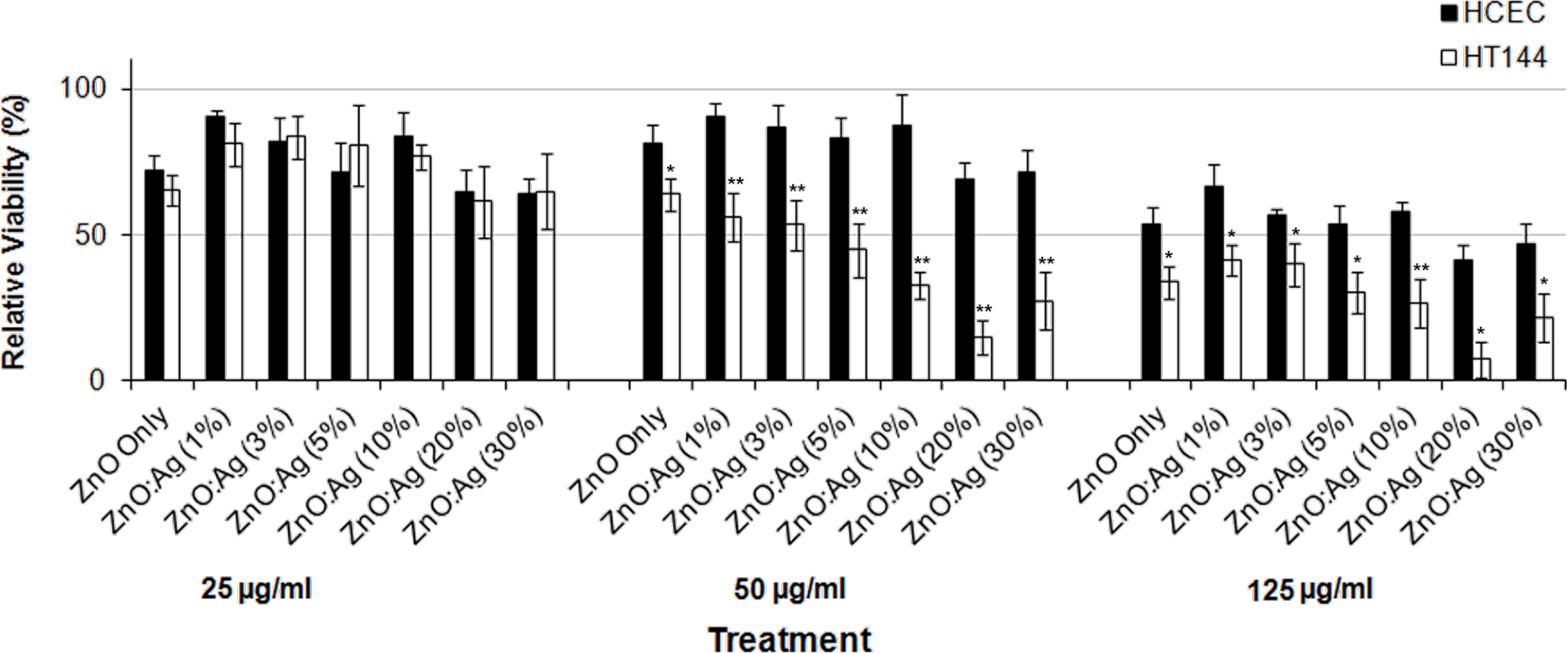
Effect of ZnO and ZnO:Ag nanocomposites on the viability of HT144 (skin cancer) and HCEC (normal) cells using SRB assay. Exponentially growing cultures were treated with different concentrations of the nanoparticles (25, 50, and 125 µg/mL) for 24 h. Percent viabilities (mean ± SD) were calculated relative to NTC. *p < 0.001, **p < 0.0001 (two tailed t-test) when compared to HCEC.

The ZnO:Ag nanocomposites were screened for cytotoxicity against two cell lines, HT144 (human malignant melanoma) and HCEC (normal cell line). The cells were treated with different dilutions of the NPs ranging from 0 to 125 μg/mL for 24 h and then submitted to the SRB assay. NP treatments were categorized as having ‘no effect’ when the measured viability was above 50%, having a ‘moderate effect’ when the viability was between 50 and 25%, and having a ‘strong effect’ when a viability below 25% was observed. By using these categories, as shown in Figure 4, ZnO NPs had ‘no differential effect’ on both normal as well as cancer cells at 25 and 50 μg/mL, however at 125 μg/mL, a moderate effect of the NPs was observed on HT144 cells.

For ZnO:Ag nanocomposites, at 25 μg/mL either ‘no effect’ was observed or the effects were similar in both the normal as well as the cancer cells and were therefore considered as not differentially affecting the cancer cells. At 50 μg/mL, a differential effect of the NPs was observed, which increased with the increase in Ag content in the NPs. ZnO:Ag (10%), ZnO:Ag (20%) and ZnO:Ag (30%) had a strong differential activity against the cancer cells as compared to the normal cells with a percent viability of 32.69 ± 4.81 μg/mL, 15.01 ± 5.85 μg/mL and 27.18 ± 9.81 μg/mL, respectively. ZnO:Ag (5%) had a slight but significant effect (*p* ≤ 0.0001) on the cancer cell line when compared to the normal cells. Whereas ZnO, ZnO:Ag (1%), ZnO:Ag (3%), had no effect on the two cell lines. At 125 μg/mL, all the NPs were significantly toxic (*p* ≤ 0.01) to HT144 as well as HECE cells when compared to the untreated cells (NTC). Hence, this concentration was considered as ‘too toxic’ for the cultures.

Depending on strongest differential effects three NPs i.e., ZnO:Ag (10%), ZnO:Ag (20%) and ZnO:Ag (30%) were selected for further analyses and ZnO NPs were included as Ag-free NP control.

### Selected NPs show strong and differential photo-oxidation mediated cytotoxicity in cancer versus normal cells

**Figure 5:**
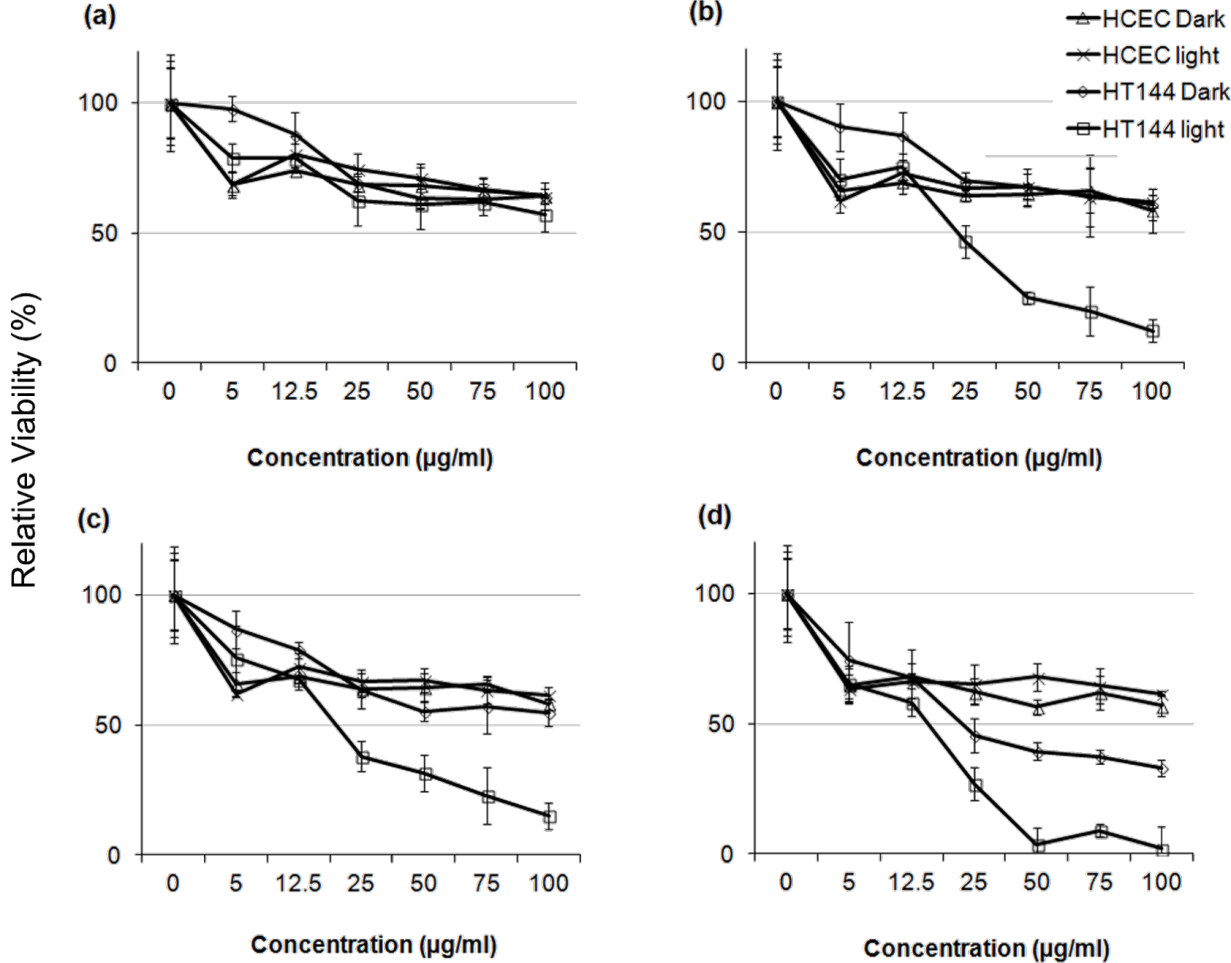
Comparison of the effect of ZnO and ZnO:Ag nanocomposites on mitochondrial function (MTT reduction) in HT144 (skin cancer) and HCEC (normal) cells. Exponentially growing cultures were treated with different concentrations (5, 12.5, 25, 50, 75, 100 µg/mL) of the nanoparticles and in order to analyze the photo-oxidative effect of nanoparticles, the cultures were exposed to daylight or kept in dark at 37 °C for 15 min and further incubated for 24 h. MTT reduction (mean ± SD) was measured. NTC were included as control. (a) ZnO, (b) ZnO:Ag (10%), (c) ZnO:Ag (20%), and (d) ZnO:Ag (30%). The experiment was performed twice with triplicates of each sample.

HT144 and HCEC cultures were exposed to increasing concentrations of the selected NPs, either exposed to daylight or kept in dark and further incubated for 24 h. Percent viabilities were calculated relative to NTC and IC_50_ values were calculated from the drug response curves (Figure 5). ZnO did not kill more than 50% (IC_50_ ≥ 100 μg/mL) of the HT144 and HCEC cells at the concentrations tested. ZnO:Ag (10%) and ZnO:Ag (20%) did not kill more than 50% of the HCEC cells (IC_50_ ≥ 100 μg/mL) at the concentrations tested under both light and dark conditions. For HT144 these NPs did not kill more than 50% of the cells at the concentrations tested under dark conditions. However, samples that were exposed to light showed a marked sensitivity to the nanocomposites with an IC_50_ of 23.37 and 19.95 μg/mL, respectively. ZnO:Ag (30%) treated HT144 cells were highly sensitive to the NPs under both light and dark conditions, with an IC_50_ of 15.78 and 23.34 μg/mL, respectively. Complete cell death was observed at 50, 75 and 100 μg/mL under light exposure. However, it did not kill more than 50% of the HCEC cells (IC_50_ ≥ 100 μg/mL) at the concentrations tested under both light and dark conditions.

The results indicate that ZnO:Ag nanocomposites have a higher photo-oxidative effect when compared with ZnO particles. In addition, the selected NPs show strong and differential cytotoxicity in cancer versus normal cells.

### Detection of singlet oxygen by chemical trapping (DPBF)

The release of ^1^O_2_ into aqueous solution was estimated indirectly by using the DPBF assay. DPBF reacts irreversibly with ^1^O_2_ causing a decrease in its absorption intensity at 410 nm. The different NPs (100 µg/mL) were mixed in DPBF solution and upon irradiation absorption was measured over a period of time. The natural logarithm values of absorption of DPBF were calculated to show an increase in the amount of ^1^O_2_.

**Figure 6:**
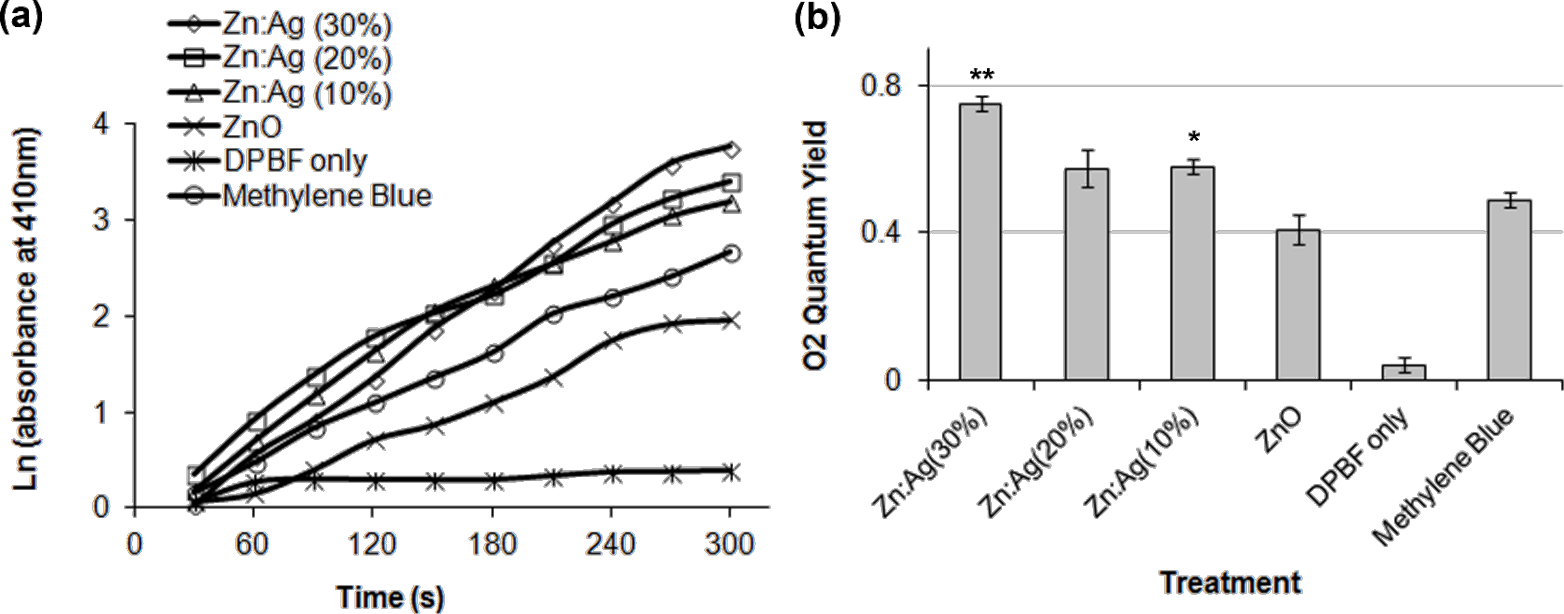
Consumptions of the singlet oxygen indicator DPBF mixed with ZnO and ZnO:Ag nanocomposites under the exposure to light and recorded every 30 s. (a) Time course of the natural log of absorption spectrum of DPBF at 410 nm. (b) Quantum yield (mean ± SD) of singlet oxygen. *p < 0.01, **p < 0.0001 (two tailed t-test) in comparison to MB.

As shown in Figure 6, the samples with only DPBF (quantum yield Φ_Δ_ = 0.043 ± 0.02) had a slight increase in the ^1^O_2_ production, which represents the baseline ^1^O_2_ release by DPBF itself whereas methylene blue (MB; Φ_Δ_ = 0.49 ± 0.02) caused a significant increase in the ^1^O_2_ levels. The ZnO NPs (Φ_Δ_ = 0.41 ± 0.04) also had a significant increase in the ^1^O_2_ levels. However, its level was slightly lower than that of MB. Both ZnO:Ag (10%) and ZnO:Ag (20%) nanocomposites (Φ_Δ_ = 0.58 ± 0.02 and Φ_Δ_ = 0.57 ± 0.05, respectively) had a similar induction of ^1^O_2_ that was significantly increased compared to the ZnO NPs. For Zn:Ag (30%) (Φ_Δ_ = 0.75 ± 0.02) a much stronger induction (*p* < 0.0001) in ^1^O_2_ production was observed and the level was about double compared to that of ZnO NPs. These results indicate that ZnO NPs induce the production of ^1^O_2_ and this production is significantly improved in ZnO:Ag nanocomposites in aqueous solution.

### Induction of oxidative stress: lipid peroxidation (LPO)

To investigate the induction of oxidative stress by the nanocomposites, cells were cultured at various concentrations (5, 12.5 and 25 µg/mL) of the NPs for 24 h, followed by the evaluation of malondialdehyde (MDA) levels by TBARs assay. As controls NTC and non-cellular background samples were also included.

**Figure 7:**
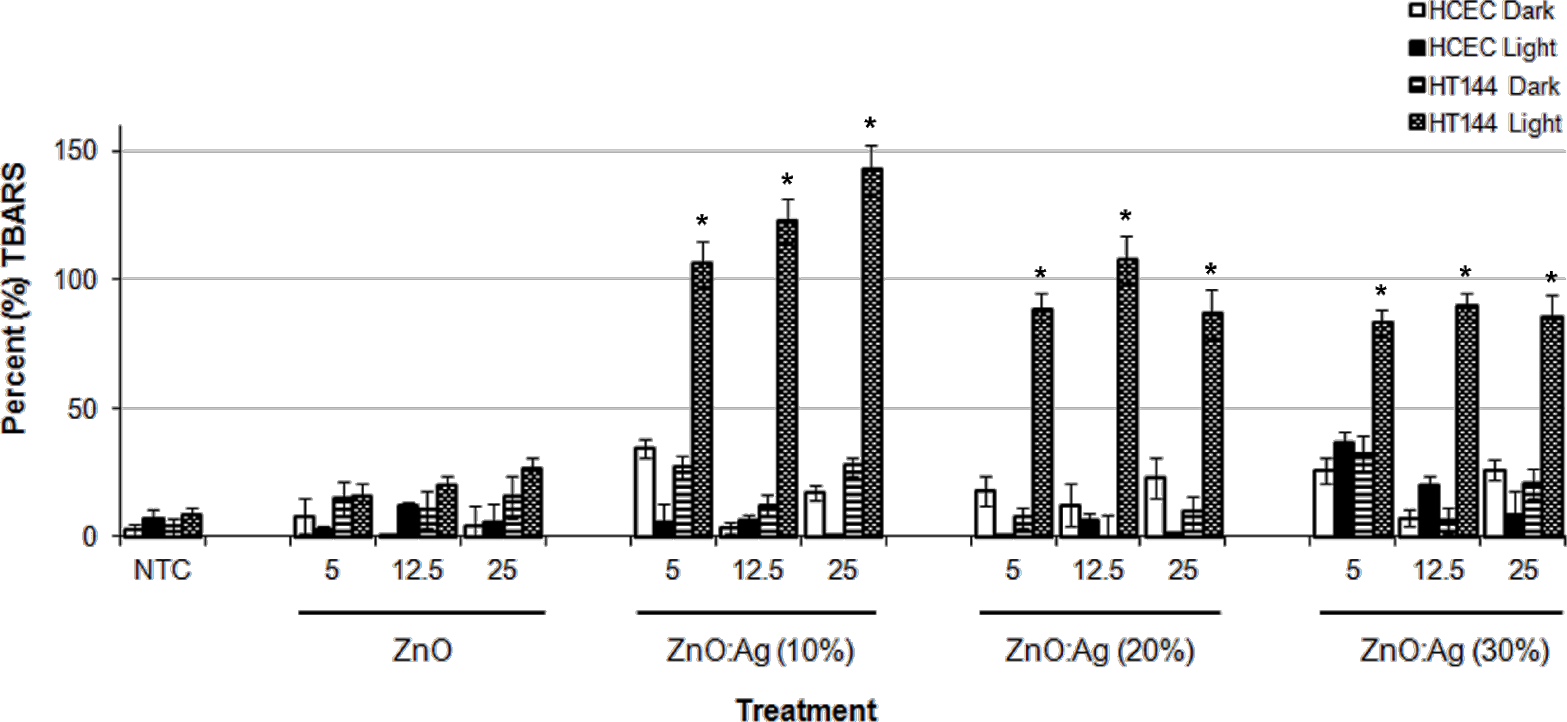
TBA assay results for nanoparticle exposure. Data is expressed as percent (%) TBARS (mean ± SD) relative to the NTC sample. *p < 0.0001 (two tailed t-test) when compared to HCEC dark, HCEC light and HT144 dark.

For HT144 cells, as shown in Figure 7, the average MDA level of NTC was 8.05 ± 3.73 and 4.16 ± 3.23 in light and dark exposed samples, respectively. ZnO:Ag (10%), ZnO:Ag (20%) and ZnO:Ag (30%) treatment resulted in an at least 6-fold increase in TBARs concentration compared with ZnO, under light exposure at the concentrations (5, 12.5 and 25 μg/mL) tested. A highly significant (*p* ≤ 0.0001) induction of MDA was obtained in HT144 under light exposure as compared to the samples kept under dark. For ZnO treated samples no significant increase in MDA levels was observed. For HCEC cells, the average MDA level of NTC was 7.24 ± 3.43 and 2.58 ± 2.54 in light and dark exposed samples, respectively. For nanoparticles treated samples no significant increase in MDA levels was observed either under light or dark condition.

### ROS species in ZnO:Ag nanocomposites induced oxidative stress in HT144 cells

**Figure 8:**
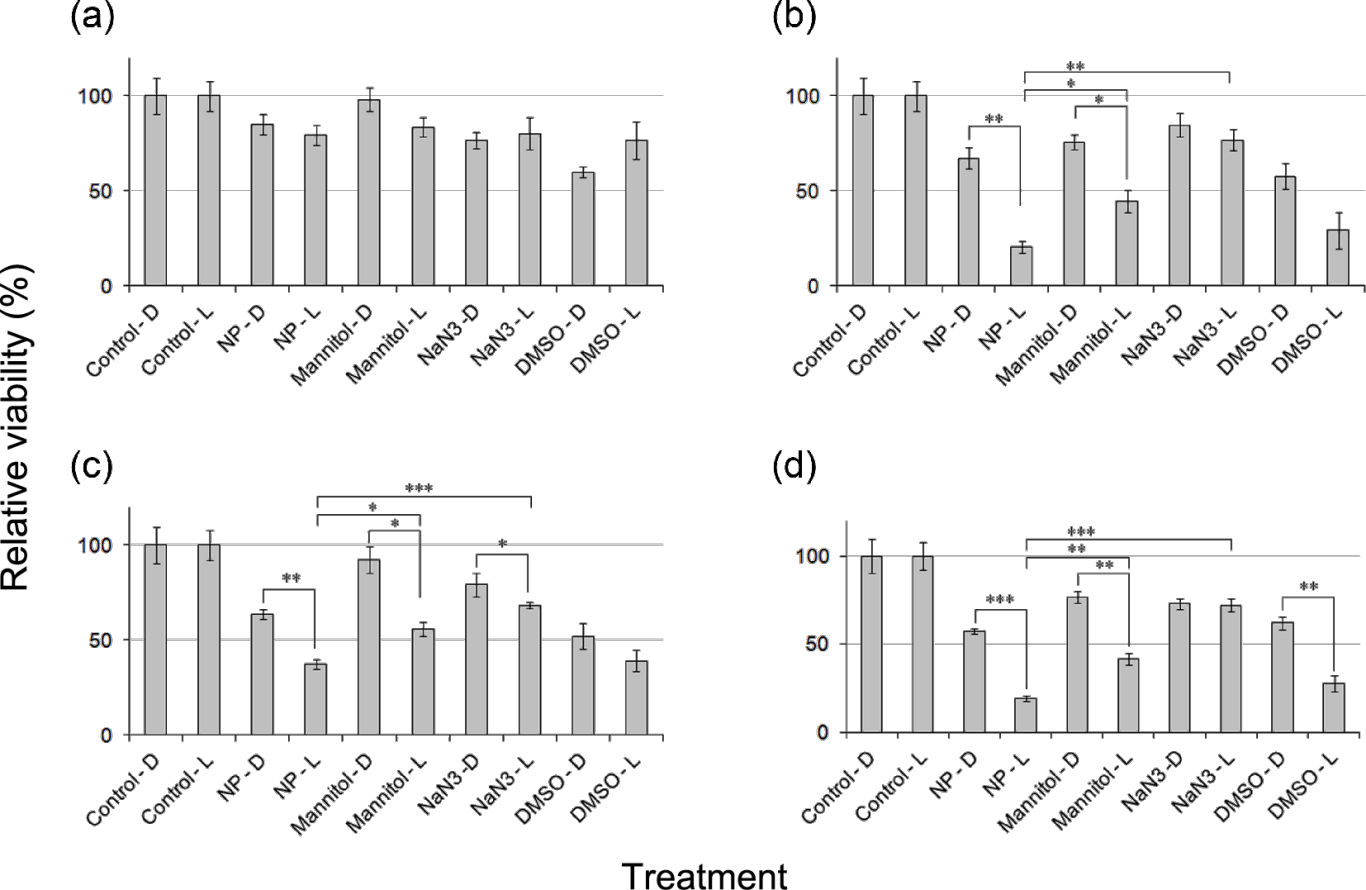
Effect of ROS scavengers mannitol, NaN_3_ and DMSO on the photo-oxidative activity of ZnO and ZnO:Ag nanocomposites as measured by MTT assay in HT144 (skin cancer) cells. Viability (mean ± SD) was calculated relative to the NTC samples. (a) ZnO, (b) ZnO:Ag (10%), (c) ZnO:Ag (20%), and (d) ZnO:Ag (30%). *p < 0.01, **p < 0.001, ***p < 0.0001 (two tailed t-test).

ROS are a family of oxygen-centered species including ^1^O_2_, HO^•^ and H_2_O_2_. To characterize the possible ROS induced by the nanocomposites, a set of ROS scavengers, namely mannitol, NaN_3_ and DMSO were used to study their inhibitory effect on NPs induced ROS formation (Figure 7). The HO^•^ scavenger mannitol improved cell viability by 10 to 30% in NP treated, light exposed samples, corroborating the involvement of HO^•^. The ^1^O_2_ scavenger NaN_3_ had a much stronger effect on cell viability and improved it by 30 to 50% in NP treated, light exposed samples, indicating ^1^O_2_ as major ROS species. DMSO addition had only a slight effect on rescuing the cells. These results demonstrate the involvement of ^1^O_2_ and HO^•^ in NPs induced ROS (Figure 8).

## Discussion

In the current study, ZnO:Ag nanocomposites with varying amounts of Ag (1, 3, 5, 10, 20, and 30%) were synthesized. As a control ZnO only NPs were also included. The nanocomposites were hexagonal in structure containing the metallic silver on surface, with a size range of 30–40 nm. The RBS analysis of ZnO:Ag nanoparticles indicated the purity of the prepared samples with the atomic percentages of Zn, O and Ag according to the expectations. Zinc and oxygen are present in correct stoichiometric amounts indicating the presence of pure ZnO and excluding any possible presence of the ZnO_2_ structure. The minor variation in oxygen amount is, however, due to oxygen vacancies created by the Fermi gas we used in our annealing procedure. These oxygen vacancies gradually obtained some atmospheric oxygen when samples were stored under normal atmospheric conditions. The nanocomposites were screened for cytotoxicity against two human cell lines, HT144 (malignant melanoma) and HCEC (normal cells). ZnO:Ag nanocomposites showed a clear photo-oxidation-mediated cytotoxic activity against the cancer cells, nanocomposites having a higher content of Ag (10 to 30%) being more toxic compared to low Ag (1 to 5%) content. ZnO NPs had no differential effect on both normal as well as cancer cells under light or dark conditions. The IC_50_ values indicate that ZnO:Ag nanocomposites have a higher cytotoxic effect when compared with ZnO NPs. In addition the selected NPs show strong and differential cytotoxicity in cancer versus normal cells. A recent study by Ismail et al. [31] reported ZnO:Ag NPs (IC_50_ values 45.10) as cytotoxic to HepG2 cells under UV illumination; however, their effect was similar to the effect of ZnO-NPs (IC_50_ values 42.60) under the same conditions. The authors however did not mention the percentage of Ag content in these NPs. Whereas our ZnO:Ag nanocomposites with a higher Ag content (10 to 30%) had a stronger and differential effect in comparison to the ZnO NPs on HT144 cells under daylight condition. The reason we have stronger effect might be due to a difference in the light source used, the percentage of Ag content or the cellular model used. Sharma et al. [32] reported zinc oxide nanoparticles with different formulations (0.1, 0.2, 0.3 and 0.4%) of Ag (size range: 23–59 nm) for their antibacterial activity and Shah et al. [33] reported that ZnO nanorods with 3% Ag content (size: 12 nm) were toxic to different bacterial strains. Talari et al. [34] reported that increase in Ag content in the ZnO:Ag nanocomposites improved the antimicrobial activity of these particles.

The addition of Ag content in ZnO NPs causes a positional shift in XRD pattern, reduction in size of the NPs and increase in the photocatalytic activity [26,34]. It is, however, not well-understood how these NPs exactly work in the exposed cells. ZnO NPs were reported to cause toxicity by generating ROS [35], causing DNA damage, oxidative stress [36], an increase in caspase-3 activity as well as p47phox NADPH (nicotinamide adenine dinucleotide phosphate)-oxidase-dependent superoxide generation [37] leading to apoptosis [21,23,38]. We therefore determine that a significantly higher amount of ^1^O_2_ was being released in the aqueous solution by ZnO:Ag (10–30%) nanocomposites, compared to the ZnO NPs under light exposure. We further investigated the induction of oxidative stress by the nanocomposites and observed a dose dependent increase in intracellular lipid peroxidation of HT144 cells only under light exposure compared to the ZnO NPs, whereas normal cells did not show such an increase. Furthermore, the study of different ROS species generated by the nanocomposites in HT144 and HCEC cells exhibited a highly significant increase in ^1^O_2_ in light exposed samples followed by HO^•^ to a lesser extent. Ismail et al. [31] also reported the production of ROS, oxidative stress and up-regulation of the antioxidant defense system as a possible mechanism for the enhanced cytotoxicity by ZnO:Ag NPs. These findings show that the incorporation of Ag in the nanocomposites substantially enhances the photo-oxidative effect and anticancer activity of the NPs. The presence of Ag significantly improves the activity of ZnO nanoparticles in visible light range thus avoiding the use of UV or infrared light.

Our findings suggest that the major mechanism by which ZnO:Ag nanocomposites mediate cell death is generation of ROS and induction of oxidative stress. ROS are involved in the activation of enzymes and transcription factors and in growth, differentiation and apoptosis [39]. The localized photo-oxidation of treated tissues affects only the area exposed to light leading to targeted tumor regression and disruption of blood supply [40]. However others have reported that ZnO NPs derived toxicity was due to dissolution of the particles and release of free metal ions leading to cell death [41–42]. It is possible that the cytotoxic effects are a result of a combination of both of these events. However, detailed investigation of the actual mode of action needs to be done.

## Conclusion

Taken together, ROS-induced oxidative damage appears to be the underlying mechanism for the anticancer activity of ZnO:Ag nanocomposites. These nanocomposites were selectively toxic to cancer cells exposed to light and at concentrations much lower than required for normal cells and quite effective compared to ZnO NP. We were able to show that the effect of ZnO NPs was improved by the formation of ZnO:Ag nanocomposites thereby improving their cell killing ability. Daylight-photodynamic therapy can provide a basis for targeted cancer treatment. However, further studies are required to evaluate the potential cytotoxicity, biocompatibility and biosafety of such particles in vitro as well as in vivo.

## Experimental

### Reagents

Acetic acid, DMSO, ethylenediaminetetraacetic acid disodium salt dihydrate (Na_2_EDTA·2H_2_O), FeSO_4_, L-glutamine, hydrochloric acid (HCl), mannitol, penicillin-G, polyethylene glycol (PEG), pyruvic acid, silver nitrate, NaN_3_, sodium chloride, sodium dodecyl sulfate (SDS), sodium hydroxide (NaOH), sodium sarcosinate, streptomycin sulfate, sulforhodamine B (SRB), 1,1,3,3-tetramethoxypropane, MTT, thiobarbituric acid (TBA), trichloroacetic acid (TCA), Triton X-100, trizma-Base, trypsin/EDTA (5%), and zinc nitrate, were purchased from Sigma-Aldrich (USA). Dulbecco's Modified Eagle Medium (DMEM) and fetal bovine serum (FBS) were purchased from GibcoBRL, Gaithersburg, MD.

### Nanocomposite synthesis

The nanocomposites ZnO:Ag (1, 3, 5, 10, 20 and 30% Ag) were synthesized following a previously reported procedure with some modifications [29]. Briefly, zinc nitrate hexahydrate and the required amount of silver nitrate (1, 3, 5, 10, 20 and 30 mol %) were dissolved in 5% v/v Tween 80 to achieve 50 mM concentration. The resulting substrate solution was titrated against 100 mM NaOH through a drop-wise addition to achieve pH 8.0. The resulting precipitates were heated at 80 °C for 2 h, and filtrated through a cellulose membrane. The resulting material was thoroughly washed, dried, ground into fine powder and sieved. The nanocomposites were heated in a tube furnace under an argon (Ar) atmosphere (flow rate 30 sccm) with a temperature rise of 4 °C/min and then kept at 100 °C for 4 h. In order to get rid of excess oxygen in the composite material, samples were further treated under reducing atmosphere (5% hydrogen in Ar) for 6 h at 450 °C (flow rate 30 sccm). Afterwards, the nanoparticles (NPs) were coated with PEG to stabilize the surface in biological environment [43]. For PEG coating, 50 mg of nanoparticles were kept stirring in a 10 mL PEG 6000 solution (1 mg/mL) for 2 h. Nanoparticles were stored in PEG environment for an extended period of time till needed for the biological evaluation. Before performing assays, we dialyzed our ZnO:Ag nanocomposite suspensions with Spectra Por 6 (mw cutoff 25000) Spectrum^®^ dialysis membrane for 4 h against deionized water.

### Characterization of nanocomposite

The nanocomposites were characterized by SEM, XRD, Rutherford back scattering, UV–vis spectroscopy and DRS analysis. The samples were gold-coated and SEM analyses were performed on a JOEL-SM6460 SEM machine. The XRD analysis of the nanocomposites was performed on Shimadzo 6000 X-ray spectrophotometer by using Cu Kα (λ = 1.54 Å) radiation at 40 kV and 30 mA. The diffraction pattern was recorded in the range of 2θ = 20–70°. Rutherford backscattering spectrometry (RBS) was carried out on a 5 MV pelletron tandem accelerator with He^++^ beam of energy 2.085 MeV employing 26 nA current and a solid state barrier detector. Detector resolution was set at 20 keV. Incident angle during analysis was kept at 0° whereas the backscattering angle was 170°. The data was analyzed via XRUMP and SIMNRA. Optical properties of composites were studied on a Lambda 950 UV–vis–NIR spectrometer. The diffused reflectance spectra were collected within a range from 900 nm to 2500 nm through an integrating sphere. Band gap energies of the nanocomposites were calculated by the Kubelka–Munk function *F*(*R*) by using the following equation [30].


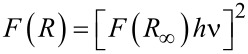


where *F*(*R*) is the Kubelka–Munk function; *R* represents the absolute value of reflectance.

### Cell lines and cell culture

Human malignant melanoma (HT144, ATCC HTB-63) cells were cultured in RPMI-1640 supplemented with 10% FBS, 2 mM L-glutamine, 1 mM Na-pyruvate, 100 U/mL penicillin, 100 µg/mL streptomycin at 37 °C in a humidified 5% CO_2_ atmosphere. The immortalized human corneal epithelial cells (HCEC; RIKEN Bio Resource Center, Japan) were cultured in DMEM containing 10% FBS, 2 mM L-glutamine, 1 mM Na-pyruvate, 100 U/mL penicillin/streptomycin at 5% CO_2_ and 37 °C in humidified environment. Cells were harvested by trypsinization with 1 mL 0.5 mM trypsin/EDTA for 1 min at room temperature.

### Screening for photo-oxidative effect of NPs

The nanoparticle stock suspensions (1 mg/mL) were prepared in de-ionized water and dispersed by sonication on sonifier cell disrupter (40 W, 25 kHz) for 30 min in a water bath. The nanoparticle serial dilutions were prepared as required by using the appropriate cell culture medium. To screen the photo-oxidation-mediated effects of the NPs, briefly, 1.5 × 10^5^ cells/mL were seeded in 25 cm^2^ flasks, 6-well plates or 96-well plates and allowed to grow over night. For the different treatments, two identical sets of samples were prepared for each cell line. One set was exposed to daylight at 37 °C for 15 min, while the second set was kept under dark at 37 °C for 15 min. Cultures were further grown at 37 °C, 5% CO_2_ for 24 h. Each experiment included NTC, media only and NPs only samples. The experiments were conducted at least twice with triplicates each.

### Measurement of cytotoxicity

Pre-seeded cells (>90% viability; 1.5 × 10^5^ cells/mL) were incubated with different dilutions of the nanoparticles (25, 50 and 125 µg/mL) for 24 h and cytotoxicity was measured by the SRB assay as previously described by Skehan et al. [44]. Briefly, cultures were fixed by gently adding 50% pre-chilled TCA and incubated at 4 °C for 1 h. The plates were rinsed with deionized water at least five times and air-dried. Samples were stained with 0.4% SRB solution for about 30 min at room temperature, rinsed with 1% acetic acid to completely remove the unincorporated dye and air-dried. The incorporated dye was solubilized in Tris (10 mM, pH 8.0) and absorbance was measured on microplate reader (AMP PLATOS R-496) at the wavelength of 565 nm.

Percent (%) viability was calculated relative to the NTC sample using the following formula:


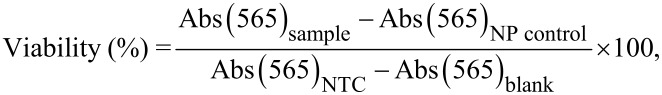


where Abs(565)_sample_ and Abs(565)_NTC_ represent the optical density at 565 nm for the treated samples and untreated control samples, respectively. Abs(565)_NP control_ and Abs(565)_blank_ represent the background optical density and was measured in NPs only and media only samples. HCEC cells were included as a normal control cell line.

### Mitochondrial function: cell survival and proliferation assay

MTT assay was used to investigate mitochondrial function as described by Mosman [45] with some modifications. In brief, pre-seeded cells (>90% viability; 1.5 × 10^5^ cells/mL) were incubated with different dilutions of the nanoparticles (5, 12.5, 25, 50, 75 and 100 µg/mL) for 24 h. As controls, NTC and the non-cellular background samples, i.e., media only and NPs only samples containing MTT and acidified 10% SDS solution were also included in the experiment. Afterwards MTT solution (0.5 mg/mL) was added and samples were incubated for 3 hours at 37 °C. The resulting formazan product was dissolved by adding equal amount of acidified 10% SDS and further incubated overnight at 37 °C. Absorbance was measured at 565 nm by using a microplate reader (AMP PLATOS R-496). The non-cellular background was subtracted from the respective samples and percent viability was measured relative to the NTC samples. Viability curves for the different nanoparticles were generated and their IC_50_ values were calculated. The IC_50_ value for the nanoparticles represents the concentration that inhibits 50% of cell growth. The experiments were performed twice with triplicates for each sample.

### Detection of singlet oxygen by chemical trapping

1,3-Diphenylisobenzofuran (DPBF) was used to determine the release of singlet oxygen (^1^O_2_) into the solution by the nanoparticles as described previously [30,43,46]. The samples were prepared immediately before use. In a typical experiment, 2 mL of an ethanol solution containing 0.08 mM DPBF and 100 μg/mL of the nanoparticles or MB solution were taken in a quartz cuvette in the dark. DPBF-only samples were also included. The experiments were carried out by exposing the samples to daylight filtered through a shortpass infrared filter (<550 nm). The absorbance of the solution was measured at 410 nm, every 30 s for 5 min with a NanoDrop 2000 (Thermo Fisher Scientific). The decrease of absorbance caused by photobleaching of DPBF was measured and corrected in all experiments. The natural logarithm values of absorption of DPBF were plotted against the irradiation time and fit by a first-order linear least-squares model to get the decay rate of the photosensitized process. The ^1^O_2_ quantum yield of the nanoparticles in aqueous solution was calculated using MB as a standard by the following formula:


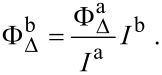



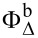
 is the ^1^O_2_ quantum yield of the nanoparticles, 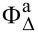 is the ^1^O_2_ quantum yield of MB that was calculated by using Rose Bengal (RB) as a standard (Φ_RB_ = 0.75 in H_2_O [47]), *I*^b^ is the slope of the nanoparticles and represents the time for the decrease in absorption of DPBF in the presence of the nanoparticles and *I*^a^ is the slope of MB and represents the time for the decrease in absorption of DPBF in the presence of the MB.

### Induction of oxidative stress

The extent of membrane lipid peroxidation (LPO) was estimated by measuring the formation of MDA by using the TBARs assay as described by Ohkawa et al. [48] with some modifications. MDA is one of the products of membrane LPO. Briefly, pre-seeded cultures (>90% viability; 1.5 × 10^5^ cells/mL) were exposed to different concentrations (5, 12.5 and 25 µg/mL) of the nanoparticles for 24 h. NTC and non-cellular background (media only and compounds only samples containing cell lysis buffer, 10% SDS and TBA solution) were included in each experiment as controls. After the treatment, cells were washed and harvested in ice-cold phosphate buffer saline at 4 °C. Cells were then lysed in cell lysis buffer (2.5 M NaCl, 10 mM Trizma-base at pH 10.0, 100 mM Na_2_EDTA, 1% sodium sarcosinate, 1% Triton X-100, 10% DMSO) and centrifuged at 15000*g* for 10 min at 4 °C. The supernatant was collected and maintained on ice until it was further assayed. To the cell lysate (0.1 mL) an equal amount of 10% SDS was added and samples were incubated at room temperature for 5 min. Then, 0.25 mL of TBA (5.2 mg/mL) was added to the mixture and incubated at 95 °C for 45 min. After cooling to room temperature, absorbance of the mixture was measured at 532 nm by NanoDrop 2000 (Thermo Fisher Scientific). The non-cellular background was subtracted from the respective samples and percent TBARs were calculated relative to the NTC sample. The experiments were performed twice with triplicates for each sample.

To study the possible ROS produced, the NPs were characterized by using a set of chemical scavengers, i.e., NaN_3_, mannitol, and DMSO. NaN_3_, a scavenger of singlet oxygen (^1^O_2_), was used at a concentration of 0.1%. Mannitol, a scavenger of hydroxyl radical (HO^•^), was used at a concentration of 10 mM (final 1 mM). DMSO, a scavenger of hydroxyl radical (HO^•^), was used at a concentration of 0.5%. Cultures were treated with the scavengers for 1 h at 37 °C before exposure to the different concentrations (5, 12.5 and 25 µg/mL) of the NPs. Cells were further incubated for 24 h at 37 °C and 5% CO_2_ and their viability was measured relative to the NTC by using MTT assay.

### Statistical analysis

The results are presented as mean ± SD. ANOVA and two tailed t-test were used to investigate the differences between NTC, nanoparticles treated and solvent exposed samples. The photo-oxidative effect of the NPs was measured by comparing the samples exposed to the different light conditions. Obtained results were considered significant when *p* < 0.05.
